# Ethyl (2*Z*)-3-oxo-2-(3,4,5-tri­meth­oxy­benzyl­idene)butano­ate

**DOI:** 10.1107/S1600536813023374

**Published:** 2013-08-23

**Authors:** Julio Zukerman-Schpector, Camila Lury Hino, Paulo J. S. Moran, Bruno R. S. de Paula, Seik Weng Ng, Edward R. T. Tiekink

**Affiliations:** a Department of Chemistry, Universidade Federal de São Carlos, 13565-905 São Carlos, SP, Brazil; b Instituto de Química, Universidade Estadual de Campinas, CP 6154, 13083-970 Campinas, SP, Brazil; c Department of Chemistry, University of Malaya, 50603 Kuala Lumpur, Malaysia; d Chemistry Department, Faculty of Science, King Abdulaziz University, PO Box 80203 Jeddah, Saudi Arabia

## Abstract

In the title compound, C_16_H_20_O_6_, the conformation about the C=C double bond [1.344 (2) Å] is *Z*. With respect to this bond, the ketone is almost coplanar [C—C—C—O torsion angle = −179.60 (10)°] and the ester is almost perpendicular [C—C—C—O = 78.42 (13)°]. The meth­oxy substituents of the central benzene ring are either almost coplanar [C—C—O—C = 3.54 (15) and 177.70 (9)°] or perpendicular [C—C—O—C = 80.08 12)° for the central substituent]. In the crystal, the three-dimensional architecture features C—H⋯O and π–π [inter-centroid distance = 3.6283 (6) Å] inter­actions.

## Related literature

For background to the study, see: Rodrigues *et al.* (2004 ▶); Zukerman-Schpector *et al.* (2011 ▶). For the synthesis of the title compound, see: de Paula (2012 ▶).
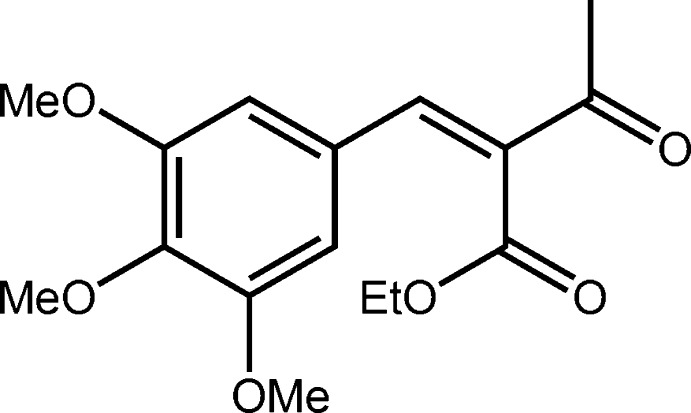


## Experimental

### 

#### Crystal data

C_16_H_20_O_6_
*M* *_r_* = 308.32
Triclinic, 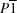

*a* = 8.3432 (4) Å
*b* = 10.2446 (5) Å
*c* = 10.4543 (5) Å
α = 61.130 (5)°
β = 77.450 (4)°
γ = 82.534 (4)°
*V* = 763.48 (7) Å^3^
*Z* = 2
Cu *K*α radiation
μ = 0.86 mm^−1^
*T* = 100 K
0.35 × 0.30 × 0.25 mm

#### Data collection

Agilent SuperNova (Dual, Cu at zero, Atlas) diffractometer
Absorption correction: multi-scan (*CrysAlis PRO*; Agilent, 2013 ▶) *T*_min_ = 0.631, *T*_max_ = 1.000
5294 measured reflections
3101 independent reflections
2943 reflections with *I* > 2σ(*I*)
*R*_int_ = 0.012

#### Refinement

*R*[*F* ^2^ > 2σ(*F* ^2^)] = 0.034
*wR*(*F* ^2^) = 0.095
*S* = 1.05
3101 reflections
204 parameters
H-atom parameters constrained
Δρ_max_ = 0.32 e Å^−3^
Δρ_min_ = −0.25 e Å^−3^

### 

Data collection: *CrysAlis PRO* (Agilent, 2013 ▶); cell refinement: *CrysAlis PRO*; data reduction: *CrysAlis PRO*; program(s) used to solve structure: *SIR92* (Altomare *et al.*, 1999 ▶); program(s) used to refine structure: *SHELXL97* (Sheldrick, 2008 ▶); molecular graphics: *ORTEP-3 for Windows* (Farrugia, 2012 ▶), *DIAMOND* (Brandenburg, 2006 ▶) and *MarvinSketch* (ChemAxon, 2010 ▶); software used to prepare material for publication: *publCIF* (Westrip, 2010 ▶).

## Supplementary Material

Crystal structure: contains datablock(s) general, I. DOI: 10.1107/S1600536813023374/kj2231sup1.cif

Structure factors: contains datablock(s) I. DOI: 10.1107/S1600536813023374/kj2231Isup2.hkl

Supplementary material file. DOI: 10.1107/S1600536813023374/kj2231Isup3.cml

Additional supplementary materials:  crystallographic information; 3D view; checkCIF report

## Figures and Tables

**Table 1:** Hydrogen-bond geometry (Å, °)

*D*—H⋯*A*	*D*—H	H⋯*A*	*D*⋯*A*	*D*—H⋯*A*
C12—H12b⋯O5^i^	0.97	2.47	3.4390 (16)	174
C14—H14a⋯O4^ii^	0.96	2.52	3.3295 (16)	142
C16—H16c⋯O2^iii^	0.96	2.55	3.4863 (15)	165

Symmetry codes: (i) 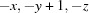; (ii) 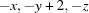; (iii) 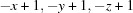.

**Fig. 1.**
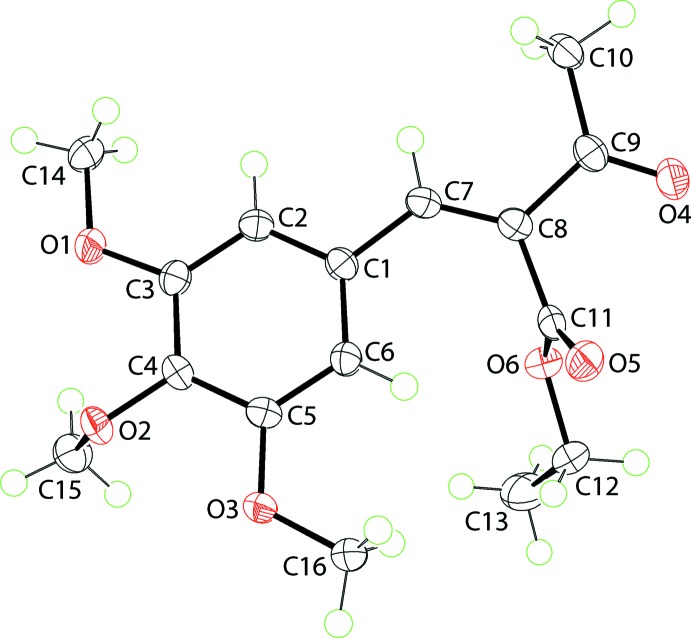
The molecular structure of compound (I) showing the atom-labelling scheme and displacement ellipsoids at the 70% probability level.

**Fig. 2.**
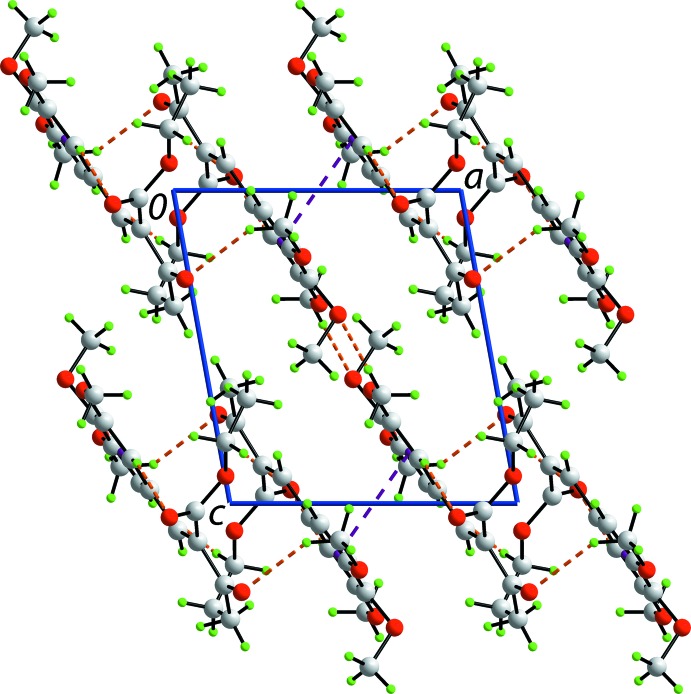
View in projection down the b axis of the unit-cell contents of (I). The π—π and C—H···O interactions are shown as purple and orange dashed lines, respectively.

## supplementary crystallographic information

### 1. Comment

As part of the continuing interest in the bio-reduction of α-haloketones and enones (Rodrigues *et al.*, 2004; Zukerman-Schpector *et al.*, 2011), the title compound, (I), was synthesized by means of a Knoevenagel condensation reaction between ethylacetoacetate and 3,4,5-trimethoxybenzaldehyde to afford a mixture of *E* and *Z* isomers that were separated by column chromatography (hexane/ethyl acetate, gradient from pure hexane to 95% hexane/5% ethyl acetate). The crystallized isomer, obtained by slow evaporation from a dichloromethane/hexane mixture, was shown by X-ray crystallography to be the *Z* isomer.

In (I), Fig. 1, the conformation about the C7═C8 bond [1.3444 (15) Å] is *Z*. The O1–O3 methoxy groups are approximately planar, perpendicular and co-planar, respectively, with the benzene ring to which they are attached as seen in the C2—C3—O1—C14, C3—C4—O2—C15 and C4—C5—O3—C16 torsion angles of 3.54 (15), 80.08 (12) and 177.70 (9)°, respectively. With respect to the ethylene bond, the ketone group is co-planar [C7—C8—C9—O4 = -179.60 (10)°] but the ester is almost perpendicular [C7—C8—C11—O6 = 78.42 (13)°]. With the exception of the ester-carbonyl-O5 atom, the two perpendicularly orientated groups lie to the same side of the central plane.

In the crystal packing, C—H···O, Table 1, and π—π [inter-centroid distance = 3.6283 (6) Å for symmetry operation: 1 - *x*, 2 - *y*, -*z*] combine to link molecules into a three-dimensional architecture. Globally, molecule pack in layers in an ···ABA···fashion running parallel to the (2,0,-1) lattice planes, Fig. 2.

### 2. Experimental

The description of the synthesis of compound (I) together with spectra and HRMS analyses can be found in de Paula (2012); *M*.pt: 381.2—381.7 K.

### 3. Refinement

Carbon-bound H-atoms were placed in calculated positions (C—H = 0.93 to 0.97 Å) and were included in the refinement in the riding model approximation, with *U_iso_*(H) = 1.2*U_eq_*(C) and 1.*5U*_eq_(methyl-C).

### Crystal data

**Table:**

C_16_H_20_O_6_	*Z* = 2
*M**_r_* = 308.32	*F*(000) = 328
Triclinic, *P*1	*D*_x_ = 1.341 Mg m^−^^3^
Hall symbol: -P 1	Melting point = 381.2–381.7 K
*a* = 8.3432 (4) Å	Cu *K*α radiation, λ = 1.54184 Å
*b* = 10.2446 (5) Å	Cell parameters from 3721 reflections
*c* = 10.4543 (5) Å	θ = 5.1–75.8°
α = 61.130 (5)°	µ = 0.86 mm^−^^1^
β = 77.450 (4)°	*T* = 100 K
γ = 82.534 (4)°	Irregular, colourless
*V* = 763.48 (7) Å^3^	0.35 × 0.30 × 0.25 mm

### Data collection

**Table:**

Agilent SuperNova (Dual, Cu at zero, Atlas) diffractometer	3101 independent reflections
Radiation source: fine-focus sealed tube	2943 reflections with *I* > 2σ(*I*)
Graphite monochromator	*R*_int_ = 0.012
Detector resolution: 10.4041 pixels mm^-1^	θ_max_ = 76.0°, θ_min_ = 5.1°
ω scans	*h* = −10→8
Absorption correction: multi-scan (*CrysAlis PRO*; Agilent, 2013)	*k* = −12→12
*T*_min_ = 0.631, *T*_max_ = 1.000	*l* = −13→12
5294 measured reflections	

### Refinement

**Table:**

Refinement on *F*^2^	Primary atom site location: structure-invariant direct methods
Least-squares matrix: full	Secondary atom site location: difference Fourier map
*R*[*F*^2^ > 2σ(*F*^2^)] = 0.034	Hydrogen site location: inferred from neighbouring sites
*wR*(*F*^2^) = 0.095	H-atom parameters constrained
*S* = 1.05	*w* = 1/[σ^2^(*F*_o_^2^) + (0.0524*P*)^2^ + 0.2494*P*] where *P* = (*F*_o_^2^ + 2*F*_c_^2^)/3
3101 reflections	(Δ/σ)_max_ < 0.001
204 parameters	Δρ_max_ = 0.32 e Å^−^^3^
0 restraints	Δρ_min_ = −0.25 e Å^−^^3^

### Special details

**Table:**

Geometry. All s.u.'s (except the s.u. in the dihedral angle between two l.s. planes) are estimated using the full covariance matrix. The cell s.u.'s are taken into account individually in the estimation of s.u.'s in distances, angles and torsion angles; correlations between s.u.'s in cell parameters are only used when they are defined by crystal symmetry. An approximate (isotropic) treatment of cell s.u.'s is used for estimating s.u.'s involving l.s. planes.
Refinement. Refinement of *F*^2^ against ALL reflections. The weighted *R*-factor *wR* and goodness of fit *S* are based on *F*^2^, conventional *R*-factors *R* are based on *F*, with *F* set to zero for negative *F*^2^. The threshold expression of *F*^2^ > 2σ(*F*^2^) is used only for calculating *R*-factors(gt) *etc*. and is not relevant to the choice of reflections for refinement. *R*-factors based on *F*^2^ are statistically about twice as large as those based on *F*, and *R*- factors based on ALL data will be even larger.

### Fractional atomic coordinates and isotropic or equivalent isotropic displacement parameters (Å^2^)

**Table:**

	*x*	*y*	*z*	*U*_iso_*/*U*_eq_	
O1	0.40766 (10)	1.13778 (8)	0.21253 (9)	0.01963 (18)	
O2	0.49401 (9)	0.85742 (8)	0.39679 (8)	0.01745 (18)	
O3	0.43618 (10)	0.62786 (8)	0.36141 (8)	0.01846 (18)	
O4	0.01540 (10)	0.84612 (9)	−0.28318 (9)	0.02070 (19)	
O5	0.21960 (10)	0.62520 (9)	−0.04434 (9)	0.02128 (19)	
O6	−0.00196 (9)	0.68398 (8)	0.08761 (8)	0.01854 (18)	
C1	0.27109 (12)	0.93633 (12)	0.04388 (11)	0.0146 (2)	
C2	0.29899 (13)	1.05493 (11)	0.06585 (12)	0.0157 (2)	
H2	0.2687	1.1516	0.0010	0.019*	
C3	0.37188 (13)	1.02961 (12)	0.18421 (12)	0.0155 (2)	
C4	0.41592 (12)	0.88409 (12)	0.28284 (11)	0.0150 (2)	
C5	0.38756 (13)	0.76540 (12)	0.26071 (11)	0.0150 (2)	
C6	0.31599 (13)	0.79048 (11)	0.14221 (11)	0.0154 (2)	
H6	0.2979	0.7110	0.1282	0.019*	
C7	0.19873 (13)	0.97298 (12)	−0.08589 (11)	0.0153 (2)	
H7	0.1953	1.0738	−0.1538	0.018*	
C8	0.13656 (13)	0.88232 (12)	−0.12120 (11)	0.0154 (2)	
C9	0.07048 (13)	0.93638 (12)	−0.26035 (12)	0.0165 (2)	
C10	0.07124 (14)	1.10044 (13)	−0.36880 (12)	0.0202 (2)	
H10A	−0.0035	1.1534	−0.3250	0.030*	
H10B	0.0377	1.1158	−0.4574	0.030*	
H10C	0.1800	1.1366	−0.3931	0.030*	
C11	0.12582 (13)	0.71652 (12)	−0.02458 (12)	0.0160 (2)	
C12	−0.02291 (16)	0.52685 (12)	0.19624 (13)	0.0229 (2)	
H12A	0.0831	0.4771	0.2147	0.027*	
H12B	−0.0786	0.4773	0.1600	0.027*	
C13	−0.12367 (17)	0.52221 (14)	0.33569 (14)	0.0299 (3)	
H13A	−0.0675	0.5722	0.3699	0.045*	
H13B	−0.1397	0.4203	0.4105	0.045*	
H13C	−0.2284	0.5712	0.3160	0.045*	
C14	0.35859 (15)	1.28797 (12)	0.11817 (13)	0.0206 (2)	
H14A	0.2415	1.2946	0.1251	0.031*	
H14B	0.4105	1.3162	0.0174	0.031*	
H14C	0.3909	1.3537	0.1488	0.031*	
C15	0.38159 (15)	0.85960 (13)	0.52181 (12)	0.0225 (2)	
H15A	0.3099	0.7769	0.5667	0.034*	
H15B	0.3175	0.9512	0.4887	0.034*	
H15C	0.4424	0.8522	0.5932	0.034*	
C16	0.40407 (16)	0.50278 (12)	0.34655 (13)	0.0224 (2)	
H16A	0.4662	0.5097	0.2548	0.034*	
H16B	0.2890	0.5020	0.3470	0.034*	
H16C	0.4354	0.4125	0.4279	0.034*	

### Atomic displacement parameters (Å^2^)

**Table:**

	*U*^11^	*U*^22^	*U*^33^	*U*^12^	*U*^13^	*U*^23^
O1	0.0258 (4)	0.0150 (4)	0.0227 (4)	0.0009 (3)	−0.0105 (3)	−0.0103 (3)
O2	0.0190 (4)	0.0215 (4)	0.0152 (4)	0.0011 (3)	−0.0063 (3)	−0.0103 (3)
O3	0.0262 (4)	0.0133 (4)	0.0173 (4)	0.0027 (3)	−0.0109 (3)	−0.0062 (3)
O4	0.0226 (4)	0.0245 (4)	0.0189 (4)	−0.0029 (3)	−0.0050 (3)	−0.0122 (3)
O5	0.0254 (4)	0.0182 (4)	0.0237 (4)	0.0029 (3)	−0.0066 (3)	−0.0124 (3)
O6	0.0205 (4)	0.0147 (4)	0.0180 (4)	−0.0011 (3)	−0.0032 (3)	−0.0056 (3)
C1	0.0137 (5)	0.0164 (5)	0.0140 (5)	0.0005 (4)	−0.0028 (4)	−0.0074 (4)
C2	0.0161 (5)	0.0140 (5)	0.0160 (5)	0.0013 (4)	−0.0039 (4)	−0.0062 (4)
C3	0.0153 (5)	0.0160 (5)	0.0168 (5)	−0.0009 (4)	−0.0018 (4)	−0.0093 (4)
C4	0.0142 (5)	0.0187 (5)	0.0136 (5)	0.0005 (4)	−0.0039 (4)	−0.0083 (4)
C5	0.0152 (5)	0.0146 (5)	0.0135 (5)	0.0014 (4)	−0.0030 (4)	−0.0055 (4)
C6	0.0173 (5)	0.0151 (5)	0.0158 (5)	0.0001 (4)	−0.0042 (4)	−0.0084 (4)
C7	0.0161 (5)	0.0145 (5)	0.0136 (5)	0.0017 (4)	−0.0033 (4)	−0.0056 (4)
C8	0.0156 (5)	0.0161 (5)	0.0137 (5)	0.0015 (4)	−0.0029 (4)	−0.0069 (4)
C9	0.0148 (5)	0.0211 (5)	0.0145 (5)	0.0008 (4)	−0.0023 (4)	−0.0095 (4)
C10	0.0242 (6)	0.0209 (5)	0.0157 (5)	0.0013 (4)	−0.0077 (4)	−0.0075 (4)
C11	0.0183 (5)	0.0174 (5)	0.0163 (5)	0.0000 (4)	−0.0077 (4)	−0.0091 (4)
C12	0.0308 (6)	0.0144 (5)	0.0215 (6)	−0.0050 (4)	−0.0061 (5)	−0.0054 (4)
C13	0.0343 (7)	0.0240 (6)	0.0234 (6)	−0.0062 (5)	−0.0003 (5)	−0.0056 (5)
C14	0.0254 (6)	0.0139 (5)	0.0244 (6)	0.0007 (4)	−0.0072 (4)	−0.0097 (4)
C15	0.0297 (6)	0.0238 (6)	0.0158 (5)	−0.0010 (5)	−0.0029 (4)	−0.0110 (5)
C16	0.0350 (6)	0.0140 (5)	0.0201 (5)	0.0014 (4)	−0.0129 (5)	−0.0067 (4)

### Geometric parameters (Å, º)

**Table:**

C1—C2	1.3962 (15)	C11—O5	1.2068 (13)
C1—C6	1.4030 (14)	C11—O6	1.3397 (13)
C1—C7	1.4667 (14)	C12—O6	1.4605 (13)
C2—C3	1.3944 (15)	C12—C13	1.4984 (17)
C2—H2	0.9300	C12—H12A	0.9700
C3—O1	1.3586 (13)	C12—H12B	0.9700
C3—C4	1.3995 (14)	C13—H13A	0.9600
C4—O2	1.3765 (12)	C13—H13B	0.9600
C4—C5	1.3993 (15)	C13—H13C	0.9600
C5—O3	1.3649 (12)	C14—O1	1.4332 (13)
C5—C6	1.3898 (15)	C14—H14A	0.9600
C6—H6	0.9300	C14—H14B	0.9600
C7—C8	1.3444 (15)	C14—H14C	0.9600
C7—H7	0.9300	C15—O2	1.4418 (13)
C8—C9	1.4893 (14)	C15—H15A	0.9600
C8—C11	1.5022 (14)	C15—H15B	0.9600
C9—O4	1.2224 (14)	C15—H15C	0.9600
C9—C10	1.5066 (15)	C16—O3	1.4274 (13)
C10—H10A	0.9600	C16—H16A	0.9600
C10—H10B	0.9600	C16—H16B	0.9600
C10—H10C	0.9600	C16—H16C	0.9600
			
C2—C1—C6	119.68 (9)	O6—C11—C8	110.49 (9)
C2—C1—C7	117.10 (9)	O6—C12—C13	106.84 (9)
C6—C1—C7	123.19 (9)	O6—C12—H12A	110.4
C3—C2—C1	120.53 (9)	C13—C12—H12A	110.4
C3—C2—H2	119.7	O6—C12—H12B	110.4
C1—C2—H2	119.7	C13—C12—H12B	110.4
O1—C3—C2	124.84 (9)	H12A—C12—H12B	108.6
O1—C3—C4	115.31 (9)	C12—C13—H13A	109.5
C2—C3—C4	119.84 (10)	C12—C13—H13B	109.5
O2—C4—C5	119.72 (9)	H13A—C13—H13B	109.5
O2—C4—C3	120.69 (9)	C12—C13—H13C	109.5
C5—C4—C3	119.51 (9)	H13A—C13—H13C	109.5
O3—C5—C6	123.90 (9)	H13B—C13—H13C	109.5
O3—C5—C4	115.35 (9)	O1—C14—H14A	109.5
C6—C5—C4	120.74 (10)	O1—C14—H14B	109.5
C5—C6—C1	119.69 (10)	H14A—C14—H14B	109.5
C5—C6—H6	120.2	O1—C14—H14C	109.5
C1—C6—H6	120.2	H14A—C14—H14C	109.5
C8—C7—C1	129.55 (10)	H14B—C14—H14C	109.5
C8—C7—H7	115.2	O2—C15—H15A	109.5
C1—C7—H7	115.2	O2—C15—H15B	109.5
C7—C8—C9	123.31 (10)	H15A—C15—H15B	109.5
C7—C8—C11	123.66 (9)	O2—C15—H15C	109.5
C9—C8—C11	113.03 (9)	H15A—C15—H15C	109.5
O4—C9—C8	118.94 (10)	H15B—C15—H15C	109.5
O4—C9—C10	121.50 (9)	O3—C16—H16A	109.5
C8—C9—C10	119.56 (9)	O3—C16—H16B	109.5
C9—C10—H10A	109.5	H16A—C16—H16B	109.5
C9—C10—H10B	109.5	O3—C16—H16C	109.5
H10A—C10—H10B	109.5	H16A—C16—H16C	109.5
C9—C10—H10C	109.5	H16B—C16—H16C	109.5
H10A—C10—H10C	109.5	C3—O1—C14	117.31 (8)
H10B—C10—H10C	109.5	C4—O2—C15	112.44 (8)
O5—C11—O6	124.60 (10)	C5—O3—C16	117.32 (8)
O5—C11—C8	124.91 (10)	C11—O6—C12	116.84 (9)
			
C6—C1—C2—C3	0.35 (16)	C1—C7—C8—C11	1.85 (18)
C7—C1—C2—C3	−178.10 (9)	C7—C8—C9—O4	−179.60 (10)
C1—C2—C3—O1	178.28 (9)	C11—C8—C9—O4	0.17 (14)
C1—C2—C3—C4	−0.69 (16)	C7—C8—C9—C10	−0.22 (16)
O1—C3—C4—O2	−1.71 (14)	C11—C8—C9—C10	179.55 (9)
C2—C3—C4—O2	177.36 (9)	C7—C8—C11—O5	−101.11 (13)
O1—C3—C4—C5	−178.50 (9)	C9—C8—C11—O5	79.13 (13)
C2—C3—C4—C5	0.57 (16)	C7—C8—C11—O6	78.42 (13)
O2—C4—C5—O3	2.47 (14)	C9—C8—C11—O6	−101.35 (10)
C3—C4—C5—O3	179.28 (9)	C2—C3—O1—C14	3.54 (15)
O2—C4—C5—C6	−176.93 (9)	C4—C3—O1—C14	−177.45 (9)
C3—C4—C5—C6	−0.11 (16)	C5—C4—O2—C15	−103.14 (11)
O3—C5—C6—C1	−179.57 (9)	C3—C4—O2—C15	80.08 (12)
C4—C5—C6—C1	−0.22 (16)	C6—C5—O3—C16	−2.92 (15)
C2—C1—C6—C5	0.11 (15)	C4—C5—O3—C16	177.70 (9)
C7—C1—C6—C5	178.46 (10)	O5—C11—O6—C12	2.89 (15)
C2—C1—C7—C8	−168.73 (11)	C8—C11—O6—C12	−176.64 (8)
C6—C1—C7—C8	12.88 (18)	C13—C12—O6—C11	157.95 (10)
C1—C7—C8—C9	−178.41 (10)		

### Hydrogen-bond geometry (Å, º)

**Table:**

*D*—H···*A*	*D*—H	H···*A*	*D*···*A*	*D*—H···*A*
C12—H12b···O5^i^	0.97	2.47	3.4390 (16)	174
C14—H14a···O4^ii^	0.96	2.52	3.3295 (16)	142
C16—H16c···O2^iii^	0.96	2.55	3.4863 (15)	165

Symmetry codes: (i) −*x*, −*y*+1, −*z*; (ii) −*x*, −*y*+2, −*z*; (iii) −*x*+1, −*y*+1, −*z*+1.
